# Comparative genomic hybridization on microarray (a-CGH) in constitutional and acquired mosaicism may detect as low as 8% abnormal cells

**DOI:** 10.1186/1755-8166-4-13

**Published:** 2011-05-09

**Authors:** Roberto Valli, Cristina Marletta, Barbara Pressato, Giuseppe Montalbano, Francesco Lo Curto, Francesco Pasquali, Emanuela Maserati

**Affiliations:** 1 Biologia e Genetica, Dipartimento di Scienze Biomediche Sperimentali e Cliniche, Università dell'Insubria, Varese, Italy

## Abstract

**Background:**

The results of cytogenetic investigations on unbalanced chromosome anomalies, both constitutional and acquired, were largely improved by comparative genomic hybridization on microarray (a-CGH), but in mosaicism the ability of a-CGH to reliably detect imbalances is not yet well established. This problem of sensitivity is even more relevant in acquired mosaicism in neoplastic diseases, where cells carrying acquired imbalances coexist with normal cells, in particular when the proportion of abnormal cells may be low.

We constructed a synthetic mosaicism by mixing the DNA of three patients carrying altogether seven chromosome imbalances with normal sex-matched DNA. Dilutions were prepared mimicking 5%, 6%, 7%, 8%, 10% and 15% levels of mosaicism. Oligomer-based a-CGH (244 K whole-genome system) was applied on the patients' DNA and customized slides designed around the regions of imbalance were used for the synthetic mosaics.

**Results and conclusions:**

The a-CGH on the synthetic mosaics proved to be able to detect as low as 8% abnormal cells in the tissue examined. Although in our experiment some regions of imbalances escaped to be revealed at this level, and were detected only at 10-15% level, it should be remarked that these ones were the smallest analyzed, and that the imbalances recurrent as clonal anomalies in cancer and leukaemia are similar in size to those revealed at 8% level.

## Introduction

The development in recent years of the microarray-based comparative genomic hybridization (a-CGH) to investigate unbalanced chromosome anomalies, both constitutional and acquired, has largely changed and improved cytogenetic investigations. Examples of application of a-CGH which revealed to be instrumental in reaching novel acquisitions are the following. Chromosome imbalances were revealed in 28 out of 140 (20%) patients with normal karyotype associated with mental disability and congenital malformations, 17 de novo and 7 inherited from a parent [1]. In a cohort of 27 patients, with an apparently balanced reciprocal translocation and an abnormal phenotype, the results of a-CGH showed chromosome imbalances in 11 of them [2]. A review of a large cohort of similar patients was offered by Sagoo et al. [3], and a comprehensive survey was done by Shaffer and Bejjani [4]. As to acquired chromosome anomalies in malignancies, the efficiency of a-CGH allowed to define the detection of putative oncogenes in the gained regions of chromosome 20q, involved in the progression of colorectal adenoma to carcinoma [5], or to demonstrate a prognostic value of the gain of chromosome regions in 1q and 16q in head and neck squamous cell carcinoma [6]. This technique detected novel cryptic copy number aberrations in the bone marrow (BM) of 15% patients with acute myeloid leukaemia (AML) and with an apparent normal karyotype [7], whereas in other cohort of patients it was instrumental in defining different and complex chromosome changes [8,9]. A review of the results in haematological malignancies obtained by a-CGH and single nucleotide polymorphism (SNP) arrays is offered by Maciejewski et al. [10]. The use of a-CGH permitted also to identify individuals predisposed to malignancy having constitutional deletions or duplications of specific tumor suppressor genes [11].

A problem seldom faced is the definition of the sensitivity of a-CGH to detect chromosome imbalances when the DNA is extracted from a cell pool containing cells with normal karyotype and cells with an unbalanced anomaly. The data available in the literature on constitutional mosaicism demonstrate that a-CGH may allow the detection even of low grades of mosaicism [1]; Ballif et al. [12] tried to estimate the detectable percentage of an abnormal clone by mixing blood cells from a normal diploid male with a trisomic 21 male, while Cheung et al. [13] suggested a formula to infer the percentage of mosaicism from a-CGH results, and assessed the possibility to detect proportions of abnormal cells around 7-8%. All these authors used BAC-based arrays differently designed. In their effort to compare the results which may be obtained by means of BAC- and oligonucleotide-based a-CGH, Neill et al. [14] conclude that mosaicisms of 30% or greater may be easily detected with both methods, and that levels as low as 10% may also be detected, but only under optimal conditions; they claim that BAC-based arrays may be more sensible to reveal mosaicism and in their study three cases with a level of mosaicism of 10% were detected with BAC-based platforms, while the lowest level of mosaicism detected with oligo array was 21%. In their paper on haematological malignancies, Maciejewski et al. [10] suggest a sensitivity of 25% abnormal cells for their SNP-array system and a probable similar sensitivity for a-CGH.

The problem of the sensitivity is even more relevant in the analysis of acquired mosaicism, that is the situation in which cells carrying acquired imbalances coexist with normal cells. In AML, 22 out of 26 cases with normal karyotype were confirmed to be normal also by a-CGH, but these results did not take into account at all the sensitivity in detecting cryptic anomalies [7]. This point is particularly relevant when the proportion of abnormal cells is expected to be low, as in some cases of myelodysplastic syndrome (MDS) [9,15], in chronic myeloproliferative disorder, in monitoring minimal residual disease (MRD), or in acquired clonal anomalies in the BM of non-malignant diseases, as Shwachman-Diamond syndrome (SDS) [16].

To asses the real sensitivity of oligo-based a-CGH platforms to detect mosaicism, we report here the results of an experimental approach based on the *in vitro *construction of DNA pools with precise percentages of DNA from cells carrying well characterized unbalanced chromosomal anomalies, mixed with DNA from cells with normal karyotype.

## Materials and methods

### Patient samples

The material for the present study was offered by three patients with constitutional unbalanced chromosome anomalies revealed by QFQ-banding and fluorescent in situ hybridization (FISH):

Patient 1, male newborn, with microcephaly and other congenital anomalies, with a deletion of the long arms of chromosome 7: the karyotype was 46,XY,del(7)(q34); fibroblasts were supplied by the NIMGS Human Genetic Cell Repository at the Coriell Institute for Medical Research (Camden, NJ, USA);

Patient 2, 20-month-old female, with psychomotor delay, facial dysmorphisms, hypotonia, bilateral pes valgus, with a deletion of the long arms of chromosome 4: karyotype 46,XX,del(4)(q34.2); a lymphoblastoid cell line was used;

Patient 3, 7-year-old female, with congenital thrombocytopenia, with a pericentric inversion of chromosome 21; FISH with informative fosmid probes showed that the rearrangement was more complex, with disruption of *RUNX1 *gene and duplication of part of it including exons 5; peripheral blood cells were used.

Informed consent to this study was obtained according to the principles of the Declaration of Helsinki from patients' parents.

## Methods

**Figure 1:**
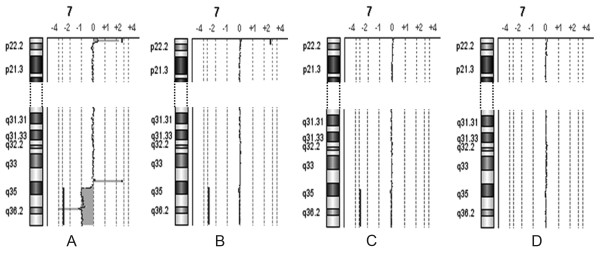
**a-CGH partial profiles of chromosome 7 in patient 1**. A) Patient's 100% DNA. B) Synthetic mosaicism at 10% level, C) 8%, D) 7%.

**Figure 2:**
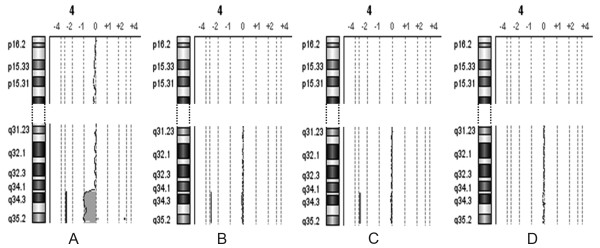
**a-CGH partial profiles of chromosome 4 in patient 2**. A) Patient's 100% DNA. B) Synthetic mosaicism at 10% level, C) 8%, D) 7%.

**Figure 3:**
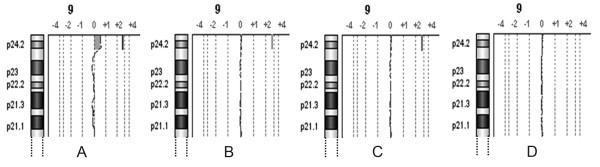
**a-CGH partial profiles of chromosome 9 in patient 2**. A) Patient's 100% DNA. B) Synthetic mosaicism at 10% level, C) 8%, D) 7%.

**Figure 4:**
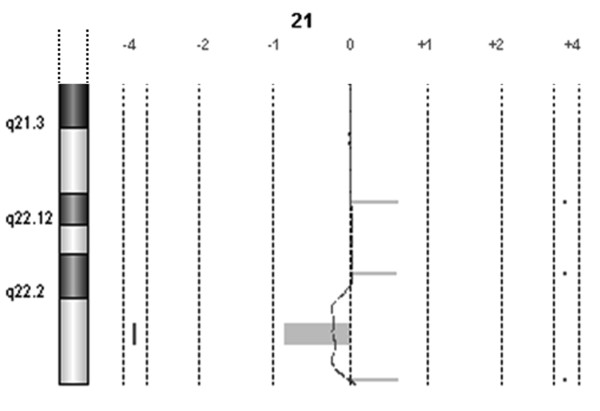
**a-CGH partial profile of chromosome 21 in patient 3**. The regions of imbalance in 21q of patient's 100% DNA.

The chromosome imbalances present in the patients were better defined by a-CGH with the whole-genome platform 244 K (Agilent Technologies, Santa Clara, CA, USA) according to the manufactory instructions (V 5.0). DNA extraction was done using the Qiagen Blood ad Tissue kit (QIAGEN GmbH, Hilden, Germany) and competitor DNA was purchased from Promega (Promega Corporation, Madison, WI, USA). Slides were scanned using Agilent's microarray scanner G2565BA and features were extracted by Agilent's Feature Extraction 9.5.1 software. The a-CGH profiles of patients were extrapolated by the Agilent's Genomic Workbench software (5.0.14), and are shown in Figures 1, 2, 3A, 4. The base pair designations from the Agilent 244 K array are according to the March 2006 Assembly (NCBI36/hg18) on the UCSC Human Genome browser http://genome.ucsc.edu/. In patient 1 the deletion of chromosome 7 was in fact terminal, with the loss of 14.164 Mb, starting at 144,657,114 bp position within the band q35 (Figure 1A). In patient 2 the a-CGH showed the terminal deletion of chromosome 4 with breakpoint in the band q34.2 with loss of 14.39 Mb, starting at 176,883,225 bp position (Figure 2A), but also that part of the terminal region of the short arms of chromosome 9 was duplicated, corresponding to a segment of 5.401 Mb (p24.1-pter), with duplication starting at 5,554,309 bp position (Figure 3A). To investigate the localization of the extra material of chromosome 9, a dual colour FISH with two BAC probes flanking the band 9p24.1 was performed and the hybridization signals showed that the extra material of chromosome 9 was transposed onto the band q34.2 of the rearranged chromosome 4, giving evidence that the anomaly was in fact an unbalanced translocation. The a-CGH results in patient 3 showed that the complex rearrangement of chromosome 21 led to four regions of imbalance of chromosome 21 (Figure 4): a 36.1 Kb duplication of part of the *RUNX1 *gene in band 22.12 (35138169-35174269 bp), a 38 Kb duplication of a segment in band 22.2 (39669148-39707107 bp), a 1.393 Mb deletion in band 22.3 (43014727-44408507 bp), and a 162 Kb duplication again in band 22.3 (46493951-46656014 bp), being the last a benign copy number variation (CNV), according to the Database of Genomic Variants, updated March 2010 [17]. So, in total, seven different unbalanced regions of different size were present in the DNA of the three patients.

We then constructed a synthetic mosaicism by mixing our patients' DNA with the same normal sex-matched DNA used as competitor. DNA concentrations (patients and reference) were first estimated by using Invitrogen's QBit fluorimeter (Invitrogen Corporation, Carlsbad, CA, USA) to obtain an absolute quantification of the samples. Then the precise relative molar ratio of the samples was assessed using Applied Biosystem's RNaseP (Applied Biosystem Corporation, Carlsbad, CA, USA) quantitative real-time PCR assay as described previously [18]. The calculated molar ratio between patient's DNA and the normal reference DNA was then used to bring all the samples to the same molar ratio. Dilutions to obtain the synthetic mosaicism were prepared at 5%, 6%, 7%, 8%, 10% and 15% of the three patients' DNA in the reference sex-matched DNA.

The a-CGH assays on pooled DNA were performed on customized slides 4 × 44 K (Agilent) and they were carried on as detailed above. Customized arrays were designed to cover the regions of interest of chromosomes 7, 4, 9, and 21, using Agilent's e-array 5.0 software http://earray.chem.agilent.com/, and other probes mapping on different chromosomes as internal control: the design format 4 × 44 K includes 43,100 selected probes, and 2,118 standard Agilent's control probes. The slides used had a spatial resolution (average probe spacing) of 9-10 Kb, similar to that of the customary whole-genome platform 244 K. For patient 3 also another different enriched customized slide was designed with all the probes available from the Agilent's HD probe catalogue (0.7-1 Kb resolution) for the three regions of subtle duplication on chromosome 21.

## Results

The a-CGH results obtained on the mixed DNA pools, mimicking acquired low grade mosaicism of 5, 6, 7, 8, 10, 15% level, showed the possibility to detect 15% mosaicism of six out of seven imbalances tested, excluding the 32 Kb duplication in the long arms of chromosome 21 of patient 3. In no case 5% and 6% mosaicism was clearly discernible. The profiles of the chromosome 7, 4, and 9 of mosaicism at levels 7, 8, and 10% in patients 1 and 2 are illustrated in Figures 1, 2, 3 (panels B, C, D): the analysis system was able to detect these three imbalances at 8 and 10% level, and not at 7%.

**Figure 5:**
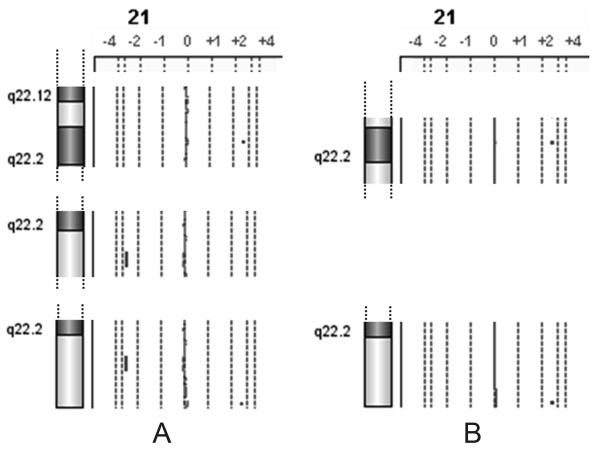
**a-CGH partial profiles of chromosome 21 in patient 3 at different levels of synthetic mosaicism**. A) 10%, B) 8%.

In patient 3, with more subtle imbalances, the results were different, depending on the fact that the imbalance is a deletion or a duplication, on its size, and on the number of oligomers covering the region involved. The a-CGH with the customized slide mimicking the 244 K resolution failed to reveal all the imbalances on chromosome 21 in diluted samples, while the customized slide enriched in probes at a resolution of 0.7-1 Kb showed: the 32 Kb duplication of part of the *RUNX1 *gene in band 22.12 was not detected, even at 15% level, the 34.4 Kb duplication in band 22.2 was detected at 10 and 8%, the 1.4 Mb deletion in band 22.3 was discerned at 10%, but not at 8%, and the 150.9 Kb duplication in band 22.3 was noticed both at 10 and 8% (Figure 5A, B).

## Discussion

The ability of a-CGH to detect reliably chromosome imbalances in mosaicism was not yet well established [14]. So, the goal of the present work was very practical: to assess the sensitivity of one of the most used oligomer-based a-CGH system to detect acquired (as well as constitutional) low level mosaicism. In particular, most recurrent unbalanced anomalies in MDS/AML and in disorders predisposing to MDS/AML have a size comparable to those of our patients 1 and 2 [19], and a-CGH may be used to monitor the abnormal clone during the disease course, and to detect MRD. Altogether our results demonstrate clearly the possibility to detect as low as 8% abnormal cells, at least for imbalances involving regions of a sufficiently great size, as those of patients 1 and 2. Obviously, more sensitive techniques are available to monitor unbalanced chromosome anomalies already identified [18,20]: our aim was not to suggest a finer method to detect them, but a solid evaluation of a-CGH sensitivity is needed to draw correct conclusions when it is used to study cohorts of patients with disorders associated with acquired chromosome anomalies, as AML [7-9], MDS [8,9,15], or diseases predisposing to MDS/AML, as Shwachman-Diamond syndrome (SDS) [16].

A technical point deserves a comment: the possibility to reach a correct result is related to the parameters of DNA quality and to the choice of an informative platform: the use of customized slides should be related to the size of the region involved and it may be necessary to design enriched slides with higher resolution probe density for the region of interest, as we did in patient 3.

In patients 1 and 2 we were able to get easily evidence of the abnormal cell population at 10 and 8% levels of mosaicism (Figures 1, 2, 3), because we had already defined the presence of the imbalance. In our material the mosaicism was built up artificially, but in general in patients in which the imbalance may be suspected from the results of a-CGH at levels comparable to those here presented, the tool to reach a conclusion is FISH with informative probes, possibly on interphase nuclei, which will be able to confirm or deny the presence of the acquired mosaicism. This comparison of a-CGH and FISH results is essential to draw definite conclusions, in particular, in case of imbalances of smaller size, where we showed that the sensitivity of a-CGH to give evidence of a small population of abnormal cells is more variable (results in patient 3) with potentially aberrant regions more difficult to be revealed: a-CGH results may in fact be really significant, and they have to be more accurately investigated. So, we suggest that whenever a-CGH indicates a possible mosaicism which may be evaluated 8-10%, as in our diluted material, FISH is crucial.

A good example of the capacity of a-CGH to detect an unexpected acquired mosaicism is offered by a patient with SDS reported in 2006 [21], who was known to have a clone with an acquired chromosome anomaly in BM, namely an isochromosome for the long arms of chromosome 7, i(7)(q10). This anomaly may be related to the risk to develop MDS/AML [16], as a deletion of the long arms of chromosome 20, del(20)(q11), another frequent change found. In the follow-up, a-CGH showed, besides the i(7)(q10), an interstitial deletion of the long arms of chromosome 20 spanning 4.116 Mb in bands q11.21-q11.23. The resolution of standard chromosome analysis was insufficient to show the deletion, but FISH with the BAC probe CTD-3092L7, mapping within the deleted region, confirmed its presence in 30/170 mitoses (17.6%), and 68/470 nuclei (14.5%) (unpublished data).

We explored also the possibility to infer the percentage of abnormal cells found in acquired mosaics by a-CGH, at least approximately. A formula derived by the ADM2 algorithm used in the analytical software permits such a calculation, and, for instance, when we applied it to a-CGH results obtained in the patient with SDS described above, led to evaluate the cell population with the deletion of chromosome 20 to be 18.2% of BM cells [22]. This evaluation agrees fairly well with the proportion of abnormal cells evaluated by FISH.

## Authors' contributions

EM, FL, and FP designed the project and wrote the manuscript. RV and CM contributed equally to a-CGH analyses. BP and GM performed chromosome analyses and FISH. All authors have read and approved the final manuscript.

## Conflict of interest

The authors declare that they have no competing interests.

## Acknoledgements

We thank the AISS (Associazione Italiana Sindrome di Shwachman), and Fondazione Banca del Monte di Lombardia.
